# Anomia is present pre-symptomatically in frontotemporal dementia due to *MAPT* mutations

**DOI:** 10.1007/s00415-022-11068-0

**Published:** 2022-03-29

**Authors:** Arabella Bouzigues, Lucy L. Russell, Georgia Peakman, Martina Bocchetta, Caroline V. Greaves, Rhian S. Convery, Emily Todd, James B. Rowe, Barbara Borroni, Daniela Galimberti, Pietro Tiraboschi, Mario Masellis, Maria Carmela Tartaglia, Elizabeth Finger, John C. van Swieten, Harro Seelaar, Lize Jiskoot, Sandro Sorbi, Chris R. Butler, Caroline Graff, Alexander Gerhard, Tobias Langheinrich, Robert Laforce, Raquel Sanchez-Valle, Alexandre de Mendonça, Fermin Moreno, Matthis Synofzik, Rik Vandenberghe, Simon Ducharme, Isabelle Le Ber, Johannes Levin, Adrian Danek, Markus Otto, Florence Pasquier, Isabel Santana, Jonathan D. Rohrer, The Genetic FTD Initiative, GENFI, Aitana Sogorb Esteve, Annabel Nelson, Arabella Bouzigues, Carolin Heller, Caroline V Greaves, David Cash, David L Thomas, Emily Todd, Hanya Benotmane, Henrik Zetterberg, Imogen J Swift, Jennifer Nicholas, Kiran Samra, Lucy L Russell, Martina Bocchetta, Rachelle Shafei, Rhian S Convery, Carolyn Timberlake, Thomas Cope, Timothy Rittman, Alberto Benussi, Enrico Premi, Roberto Gasparotti, Silvana Archetti, Stefano Gazzina, Valentina Cantoni, Andrea Arighi, Chiara Fenoglio, Elio Scarpini, Giorgio Fumagalli, Vittoria Borracci, Giacomina Rossi, Giorgio Giaccone, Paola Caroppo, Pietro Tiraboschi, Sara Prioni, Veronica Redaelli, David Tang-Wai, Ekaterina Rogaeva, Miguel Castelo-Branco, Ron Keren, Sandra Black, Sara Mitchell, Christen Shoesmith, Robart Bartha, Rosa Rademakers, Jackie Poos, Janne M Papma, Lucia Giannini, Rick Minkelen, Yolande Pijnenburg, Benedetta Nacmias, Camilla Ferrari, Cristina Polito, Gemma Lombardi, Valentina Bessi, Michele Veldsman, Christin Andersson, Hakan Thonberg, Linn Öijerstedt, Vesna Jelic, Paul Thompson, Tobias Langheinrich, Albert Lladó, Anna Antonell, Jaume Olives, Mircea Balasa, Nuria Bargalló, Sergi Borrego-Ecija, Ana Verdelho, Carolina Maruta, Catarina B Ferreira, Gabriel Miltenberger, Frederico Simões do Couto, Alazne Gabilondo, Ana Gorostidi, Jorge Villanua, Marta Cañada, Mikel Tainta, Miren Zulaica, Myriam Barandiaran, Patricia Alves, Benjamin Bender, Carlo Wilke, Lisa Graf, Annick Vogels, Mathieu Vandenbulcke, Philip Van Damme, Rose Bruffaerts, Koen Poesen, Pedro Rosa-Neto, Serge Gauthier, Agnès Camuzat, Alexis Brice, Anne Bertrand, Aurélie Funkiewiez, Daisy Rinaldi, Dario Saracino, Olivier Colliot, Sabrina Sayah, Catharina Prix, Elisabeth Wlasich, Olivia Wagemann, Sandra Loosli, Sonja Schönecker, Tobias Hoegen, Jolina Lombardi, Sarah Anderl-Straub, Adeline Rollin, Gregory Kuchcinski, Maxime Bertoux, Thibaud Lebouvier, Vincent Deramecourt, Beatriz Santiago, Diana Duro, Maria João Leitão, Maria Rosario Almeida, Miguel Tábuas-Pereira, Sónia Afonso, Annerose Engel, Maryna Polyakova

**Affiliations:** 1 Department of Neurodegenerative Disease, Dementia Research Centre, UCL Institute of Neurology, Queen Square, London, WC1N 3BG UK; 2 Trust and Medical Research Council Cognition and Brain Sciences Unit, Department of Clinical Neurosciences and Cambridge University Hospitals NHS, University of Cambridge, Cambridge, UK; 3 Centre for Neurodegenerative Disorders, Department of Clinical and Experimental Sciences, University of Brescia, Brescia, Italy; 4 Department of Biomedical, Surgical and Dental Sciences, University of Milan, Milan, Italy; 5 Fondazione IRCCS Ca’ Granda, Ospedale Maggiore Policlinico, Milan, Italy; 6 Fondazione IRCCS Istituto Neurologico Carlo Besta, Milano, Italy; 7 Sunnybrook Health Sciences Centre, Sunnybrook Research Institute, University of Toronto, Toronto, Canada; 8 Tanz Centre for Research in Neurodegenerative Diseases, University of Toronto, Toronto, Canada; 9 Department of Clinical Neurological Sciences, University of Western Ontario, London, ON Canada; 10 Department of Neurology, Erasmus Medical Centre, Rotterdam, Netherlands; 11 Department of Neurofarba, University of Florence, Florence, Italy; 12 IRCCS Fondazione Don Carlo Gnocchi, Florence, Italy; 13 Nuffield Department of Clinical Neurosciences, Medical Sciences Division, University of Oxford, Oxford, UK; 14 Department of Brain Sciences, Imperial College London, London, UK; 15 Division of Neurogeriatrics, Department of Neurobiology, Care Sciences and Society, Center for Alzheimer Research, Bioclinicum, Karolinska Institutet, Solna, Sweden; 16 Unit for Hereditary Dementias, Theme Aging, Karolinska University Hospital, Solna, Sweden; 17 Division of Neuroscience and Experimental Psychology, Wolfson Molecular Imaging Centre, University of Manchester, Manchester, UK; 18 Departments of Geriatric Medicine and Nuclear Medicine, University of Duisburg, Essen, Germany; 19 Cerebral Function Unit, Manchester Centre for Clinical Neurosciences, Salford Royal NHS Foundation Trust, Salford, UK; 20 Département Des Sciences Neurologiques, Clinique Interdisciplinaire de Mémoire, CHU de Québec, and Faculté de Médecine, Université Laval, Québec, QC Canada; 21 Alzheimer’s Disease and Other Cognitive Disorders Unit, Neurology Service, Hospital Clínic, Institut d’Investigacións Biomèdiques August Pi I Sunyer, University of Barcelona, Barcelona, Spain; 22 Faculty of Medicine, University of Lisbon, Lisbon, Portugal; 23 Cognitive Disorders Unit, Department of Neurology, Donostia University Hospital, San Sebastian, Gipuzkoa, Spain; 24 Neuroscience Area, Biodonostia Health Research Institute, Gipuzkoa, San Sebastian, Spain; 25 Department of Neurodegenerative Diseases, Hertie-Institute for Clinical Brain Research and Center of Neurology, University of Tübingen, Tübingen, Germany; 26 Center for Neurodegenerative Diseases (DZNE), Tübingen, Germany; 27 Laboratory for Cognitive Neurology, Department of Neurosciences, KU Leuven, Leuven, Belgium; 28 Neurology Service, University Hospitals Leuven, Leuven, Belgium; 29 Leuven Brain Institute, KU Leuven, Leuven, Belgium; 30 Department of Psychiatry, Douglas Mental Health University Institute, McGill University, Montreal, Canada; 31 Department of Neurology & Neurosurgery, McConnell Brain Imaging Centre, Montreal Neurological Institute, McGill University, Montreal, Canada; 32 Paris Brain Institute – Institut du Cerveau – ICM, Inserm U1127, CNRS UMR 7225, Sorbonne Université, AP-HP - Hôpital Pitié-Salpêtrière, Paris, France; 33 Centre de Référence Des Démences Rares Ou Précoces, IM2A, Département de Neurologie, AP-HP - Hôpital Pitié-Salpêtrière, Paris, France; 34 Département de Neurologie, AP-HP - Hôpital Pitié-Salpêtrière, Paris, France; 35 Neurologische Klinik Und Poliklinik, Ludwig-Maximilians-Universität, Munich, Germany; 36 Center for Neurodegenerative Diseases (DZNE), Munich, Germany; 37 Munich Cluster of Systems Neurology, Munich, Germany; 38 Department of Neurology, University of Ulm, Ulm, Germany; 39 Univ Lille, Lille, France; 40 Inserm 1172, Lille, France; 41 CHU, CNR-MAJ, Labex Distalz, LiCEND Lille, Lille, France; 42 Neurology Service, Faculty of Medicine, University Hospital of Coimbra (HUC), University of Coimbra, Coimbra, Portugal; 43 Center for Neuroscience and Cell Biology, Faculty of Medicine, University of Coimbra, Coimbra, Portugal

**Keywords:** Frontotemporal dementia, Tau, Progranulin, C9orf72, Naming, Cognition

## Abstract

**Introduction:**

A third of frontotemporal dementia (FTD) is caused by an autosomal-dominant genetic mutation in one of three genes: microtubule-associated protein tau (*MAPT*), chromosome 9 open reading frame 72 (*C9orf72*) and progranulin (*GRN*). Prior studies of prodromal FTD have identified impaired executive function and social cognition early in the disease but few have studied naming in detail.

**Methods:**

We investigated performance on the Boston Naming Test (BNT) in the GENetic Frontotemporal dementia Initiative cohort of 499 mutation carriers and 248 mutation-negative controls divided across three genetic groups: *C9orf72*, *MAPT* and *GRN*. Mutation carriers were further divided into 3 groups according to their global CDR plus NACC FTLD score: 0 (asymptomatic), 0.5 (prodromal) and 1 + (fully symptomatic). Groups were compared using a bootstrapped linear regression model, adjusting for age, sex, language and education. Finally, we identified neural correlates of anomia within carriers of each genetic group using a voxel-based morphometry analysis.

**Results:**

All symptomatic groups performed worse on the BNT than controls with the *MAPT* symptomatic group scoring the worst. Furthermore, *MAPT* asymptomatic and prodromal groups performed significantly worse than controls. Correlates of anomia in *MAPT* mutation carriers included bilateral anterior temporal lobe regions and the anterior insula. Similar bilateral anterior temporal lobe involvement was seen in *C9orf72* mutation carriers as well as more widespread left frontal atrophy. In *GRN* mutation carriers, neural correlates were limited to the left hemisphere, and involved frontal, temporal, insula and striatal regions.

**Conclusion:**

This study suggests the development of early anomia in *MAPT* mutation carriers, likely to be associated with impaired semantic knowledge. Clinical trials focused on the prodromal period within individuals with *MAPT* mutations should use language tasks, such as the BNT for patient stratification and as outcome measures.

**Supplementary Information:**

The online version contains supplementary material available at 10.1007/s00415-022-11068-0.

## Introduction

Frontotemporal dementia (FTD) is a heterogeneous neurodegenerative disorder presenting with distinct changes in behaviour, language and motor function [1]. A third of cases are caused by an autosomal-dominant genetic mutation in one of three genes: microtubule-associated protein tau (*MAPT*), chromosome 9 open reading frame 72 (*C9orf72*) and progranulin (*GRN*) [2]. Although mutations in any of these genes can lead to impaired naming ability (anomia), *MAPT* mutation carriers tend to show the most pronounced deficit with previous studies showing that such difficulties can even be detected before symptom onset [3–5]. Importantly, whilst anomia is one of the key manifestations of people with the language variant of FTD [6], a similar pattern of naming deficits, albeit often less severe, has been found in the early stages of people presenting with both behavioural and motor symptoms [7–9], suggesting that impairment could potentially be seen in all of the phenotypes of genetic FTD.

Neuroanatomical correlates of naming deficits in FTD have implicated a widespread network of brain regions focused on the left hemisphere [10], which reflects the different components of the language pathway that contribute to naming [11]. In FTD due to *C9orf72*, *GRN* or *MAPT* mutations, there are both shared and distinct networks of atrophy across genetic groups, observable even at the pre-symptomatic stage [12]. This raised our hypothesis that the neuroanatomical correlates underlying naming differ according to the genetic aetiology of FTD.

The current study assessed naming deficits using the short 30-item version of the Boston Naming Test (BNT) [13] in a large cohort of *C9orf72*, *MAPT* and *GRN* mutation carriers. We expected all symptomatic mutation carriers to be impaired compared to mutation-negative controls on the BNT, but that *MAPT* mutation carriers would be the most impaired, potentially even in pre-symptomatic stages [4]. We also aimed to investigate the neural correlates of the BNT within each genetic group using voxel-based morphometric analyses of grey matter volume derived from structural Magnetic Resonance Imaging (MRI). We expected regions of the left-lateralised language network to be implicated in naming deficits across the groups, with potentially more focal anterior medial temporal structures in *MAPT* mutation carriers and a wider network in the *C9orf72* and *GRN* groups.

## Methods

### Participants

Participants were recruited from the fifth data freeze of the GENFI study including sites in the UK, Canada, Sweden, Netherlands, Belgium, Spain, France, Portugal, Italy and Germany with eight different languages. Ethical approval was obtained for the study and all participants provided informed written consent. As well as the 30-item version of the Boston Naming Test in their preferred language [14], all participants underwent a standardised GENFI clinical assessment including a medical history, physical examination, the Mini-Mental State Examination (MMSE), and the Clinical Dementia Rating Scale (CDR) with National Alzheimer’s Coordinating Centre (NACC) FTD-specific modules (CDR plus NACC FTLD). The CDR plus NACC FTLD provides both a summed score (CDR plus NACC FTLD sum of boxes) and a global score, where 0 is asymptomatic, 0.5 is prodromal, 1 is mildly symptomatic, 2 is moderately symptomatic and 3 is severely symptomatic, with the last three scores also being combined to create a 1 + or ‘fully symptomatic’ group [15].

747 GENFI participants completed the BNT and were included in the present study: 248 mutation-negative carriers (controls), 212 *C9orf72* expansion carriers, 201 *GRN* mutation carriers, and 86 *MAPT* mutation carriers. Mutation carriers were further divided into three groups according to their CDR plus NACC FTLD global score. Within the symptomatic mutation carrier groups, 101 met the diagnostic criteria for behavioural variant FTD (bvFTD: 54 *C9orf72*, 26 *GRN* and 21 *MAPT*), 20 primary progressive aphasia (PPA: 3 *C9orf72*, 16 *GRN* and 1 *MAPT*) and 14 amyotrophic lateral sclerosis with or without FTD (14 *C9orf72*). Demographic data for the groups are described in Table 1.

**Table 1:** Demographic data showing the number of participants as well as the age, sex (percentage males) and education of each group.

	*N*	Age	% Male	Education	CDR plus NACC FTLD SOB	MMSE	BNT
Controls	248	44.9 (12.7)	43.2	14.4 (3.2)	0.0 (0.0)	29.3 (1.1)	27.8 (1.9)
C9orf72							
0	110	44.2 (11.7)	41.8	14.3 (3.0)	0.0 (0.0)	29.2 (1.1)	27.3 (3.1)
0.5	36	49.3 (11.4)	38.9	14.1 (2.5)	1.2 (0.8)	28.6 (2.0)	27.5 (3.4)
1 +	66	62.1 (8.6)	65.2	13.2 (3.7)	10.7 (5.4)	24.0 (5.8)	20.6 (7.6)
GRN							
0	128	45.8 (12.2)	35.2	14.7 (3.4)	0.0 (0.0)	29.4 (0.9)	27.9 (1.9)
0.5	30	51.7 (13.4)	50.0	14.0 (4.0)	1.0 (0.8)	28.4 (2.4)	26.7 (3.7)
1 +	43	63.5 (7.9)	51.2	11.9 (3.3)	8.6 (5.4)	21.3 (6.1)	21.2 (6.5)
MAPT							
0	48	39.3 (10.5)	39.6	14.4 (3.6)	0.0 (0.0)	29.5 (0.8)	27.6 (2.1)
0.5	14	45.7 (12.6)	28.6	13.5 (2.4)	1.1 (0.8)	28.2 (2.3)	25.7 (3.9)
1 +	24	57.3 (10.2)	66.7	13.7 (3.9)	9.3 (5.5)	23.7 (6.7)	17.0 (8.0)

CDR plus NACC FTLD sum of boxes (SOB) score is shown as well as the Mini-Mental State Examination (MMSE) and Boston Naming Test. Scores are shown as means (standard deviations)

### Magnetic Resonance Imaging (MRI)

Participants underwent volumetric T1-weighted magnetic resonance imaging (MRI) according to the harmonized GENFI imaging protocol on a 3T scanner, with only mutation carriers included in the neural correlate imaging analysis. From a total of 499 mutation carriers included in the naming study, 94 were excluded from the imaging analysis due to either imaging not being performed or not passing quality control. 405 scans were included: Siemens Trio 3T (*n* = 111), Siemens Skyra 3T (*n* = 64), Siemens Prisma 3T (*n* = 91), Philips Achieva 3T (*n* = 135) and GE 3T (*n* = 4).

### BNT statistical analysis

Statistical analyses were performed using STATA version 16.0 (Texas, USA). The significance level was set at *p* < 0.05 across all comparisons. We compared group demographic data with linear regression except for sex which was compared using chi-square tests.

BNT scores in controls were assessed by calculating cumulative frequency (and therefore percentile scores), as well as investigating the effect of sex (Mann–Whitney *U* test), age (Spearman’s rank correlation), and education (Spearman’s rank correlation).

BNT scores in the mutation carrier groups were compared to each other and to controls using a bootstrapped linear mixed effects model (2000 repetitions) (due to non-normality). The model was adjusted for age, sex, education, language and family clustering with 95% bootstrapped confidence intervals. Post hoc pairwise comparisons were used to assess differences in group performance.

### Structural brain imaging analysis

Voxel-based morphometric (VBM) analysis was performed using Statistical Parametric Mapping (SPM) 12 software, version 7219 (www.fil.ion.ucl.ac.uk/spm), running under Matlab R2014b (Mathworks, USA). The T1-weighted images were normalized and segmented into grey matter (GM), white matter (WM) and cerebrospinal fluid (CSF) probability maps, using standard procedures and the fast-diffeomorphic image registration algorithm (DARTEL) [16]. GM segmentations were affine-transformed into the Montreal Neurological Institute (MNI) space, modulated and smoothed using a Gaussian kernel with 6 mm full width, at half maximum, before analysis. Finally, a customised explicit brain mask was applied based on an optimised voxel threshold intensity criterion [17]. All segmentations were visually checked at each stage. Total intracranial volume was calculated using SPM [18].

The relationship of BNT score with GM density in the three mutation carrier groups was explored using a flexible factorial regression model. A main effect of BNT was included in the model and genetic group was included as an interaction. Age, sex, TIV and scanner type were included as covariates in the initial model with a further model additionally including disease severity as measured by the CDR plus NACC FTLD sum of boxes. All comparisons were adjusted for multiple comparisons by applying a Family-Wise Error correction set at *p* < 0.05. An uncorrected threshold of *p* < 0.001 was used if no results were found after correcting for multiple comparisons. An empirically determined cluster size threshold was also applied (23 for the initial model, and 62 for the further model).

## Results

### Demographic data

Differences between groups were seen in age, sex and years of education (Table 1). Compared with controls, all symptomatic groups (*p* < 0.001) as well as prodromal *C9orf72* (*p* = 0.033) and *GRN* (*p* = 0.010) mutation carriers were significantly older, whilst asymptomatic *MAPT* mutation carriers were significantly younger than controls (*p* = 0.001). Within each genetic group, all symptomatic groups were significantly older than the prodromal groups (*p* < 0.003) who were significantly older than the asymptomatic groups (*p* < 0.033) apart from in *MAPT* mutations carriers where no difference in age was observed between prodromal and asymptomatic groups. There were significantly more males than females in the symptomatic *C9orf72* (*p* = 0.001) and *MAPT* (*p* = 0.027) mutation carriers compared with the control group. With genetic groups, there were significantly more males than females in the symptomatic *C9orf72* mutation carriers compared with the prodromal (*p* = 0.011) and asymptomatic (*p* = 0.003) groups. There were also more males than females in the symptomatic *MAPT* mutation carriers compared to the prodromal (*p* = 0.023) and asymptomatic (*p* = 0.030) groups. In terms of years of education, symptomatic *GRN* and *C9orf72* mutation carriers had significantly fewer years of education than controls (*p* < 0.001, *p* < 0.05). Within genetic groups, symptomatic *GRN* mutation carriers had fewer years of education compared with the other two groups (*p* < 0.05) and symptomatic *C9orf72* mutation carriers had significantly fewer years of education than the asymptomatic group (*p* < 0.05).

### BNT scores in controls

Calculation of cumulative frequency in controls revealed a 5th percentile cut-off score at 24 (Supplementary Table S1). BNT scores did not correlate with age (rho = − 0.04, *p* = 0.53), and there was no significant effect of sex on BNT score (U = − 10,590, *p* = 0.72) (Supplementary Table S2). However, there was a weak positive correlation with education (rho = 0.28, *p* < 0.001).

### BNT scores in genetic groups

**Fig. 1:**
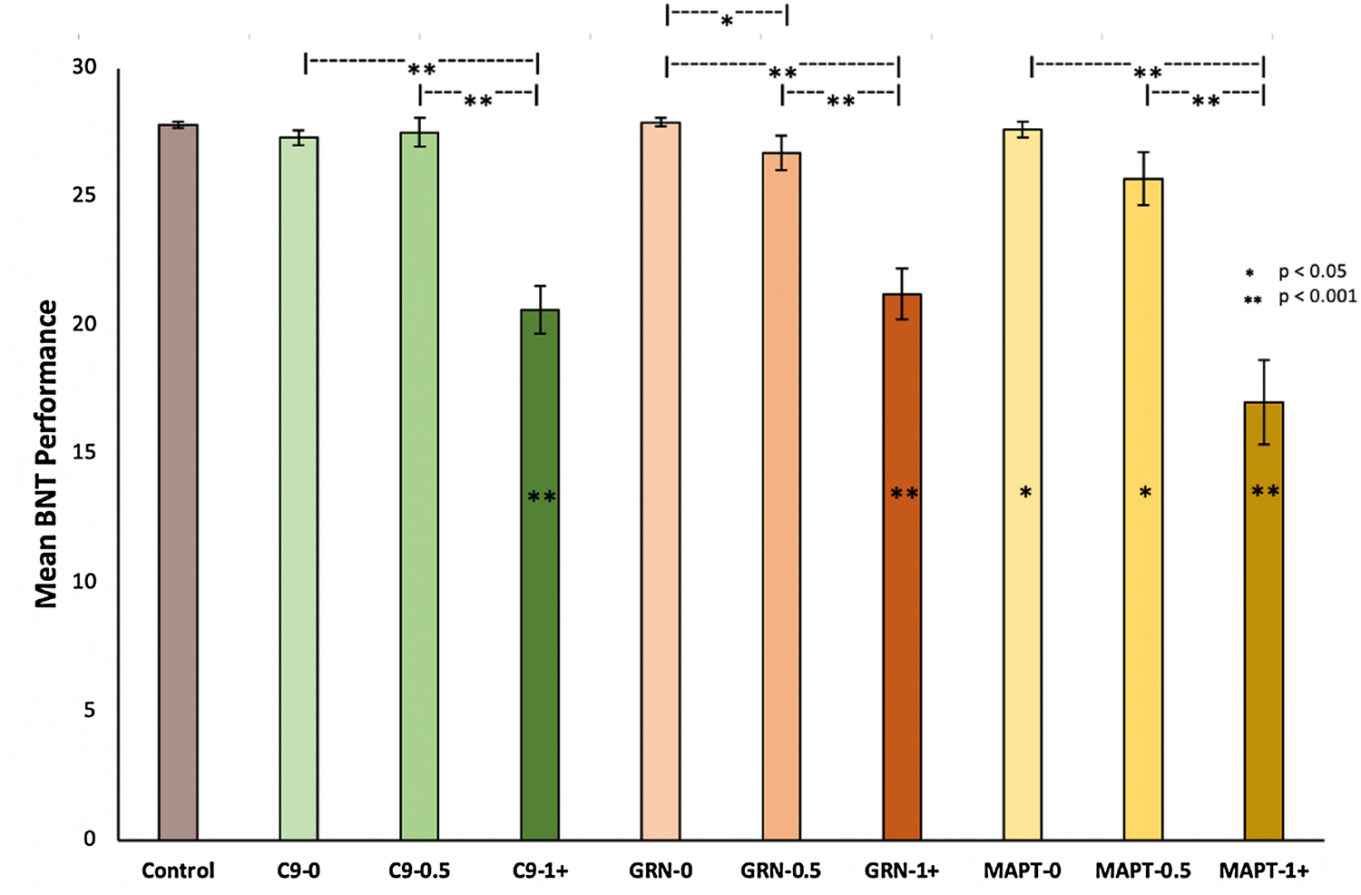
Mean scores and standard error on the BNT for each group. Significantly worse performance compared with controls is shown with a star in the bar. Only differences between disease groups and controls, and within each genetic group are shown on the graph. Additional between genetic group differences were seen between MAPT 1 + and both GRN and C9orf72 1 + , between MAPT 0.5 and C9orf72 0.5, and between both MAPT 0 and C9orf72 0 and GRN 0

All three fully symptomatic mutation carrier groups performed significantly worse than controls on the BNT (all *p* =  < 0.001) (Fig. 1, Table 1, Supplementary Table S3). Asymptomatic and prodromal *MAPT* mutation carriers also performed significantly worse than controls (*p* = 0.012 and 0.011 respectively) but neither of the *GRN* or *C9orf72* pre-symptomatic groups performed significantly worse than controls on the task.

Within genetic groups, the fully symptomatic groups performed worse than both the prodromal and asymptomatic groups in *MAPT*, *GRN* and *C9orf72* mutation carriers (all *p* =  < 0.001). Additionally, the *GRN* prodromal group scored significantly worse than the asymptomatic group (*p* = 0.018).

Between genetic groups at the same disease stage, symptomatic *MAPT* mutation carriers performed significantly worse than symptomatic *GRN* and *C9orf72* mutation carriers (*p* = 0.007 and 0.034 respectively). Prodromal *MAPT* mutation carriers performed significantly worse than prodromal *C9orf72* mutation carriers (*p* = 0.020), whilst both asymptomatic *MAPT* and *C9orf72* mutation carriers performed significantly worse than asymptomatic *GRN* carriers (*p* = 0.003, *p* = 0.048 respectively).

### Neuroanatomical correlates of BNT score

**Fig. 2:**
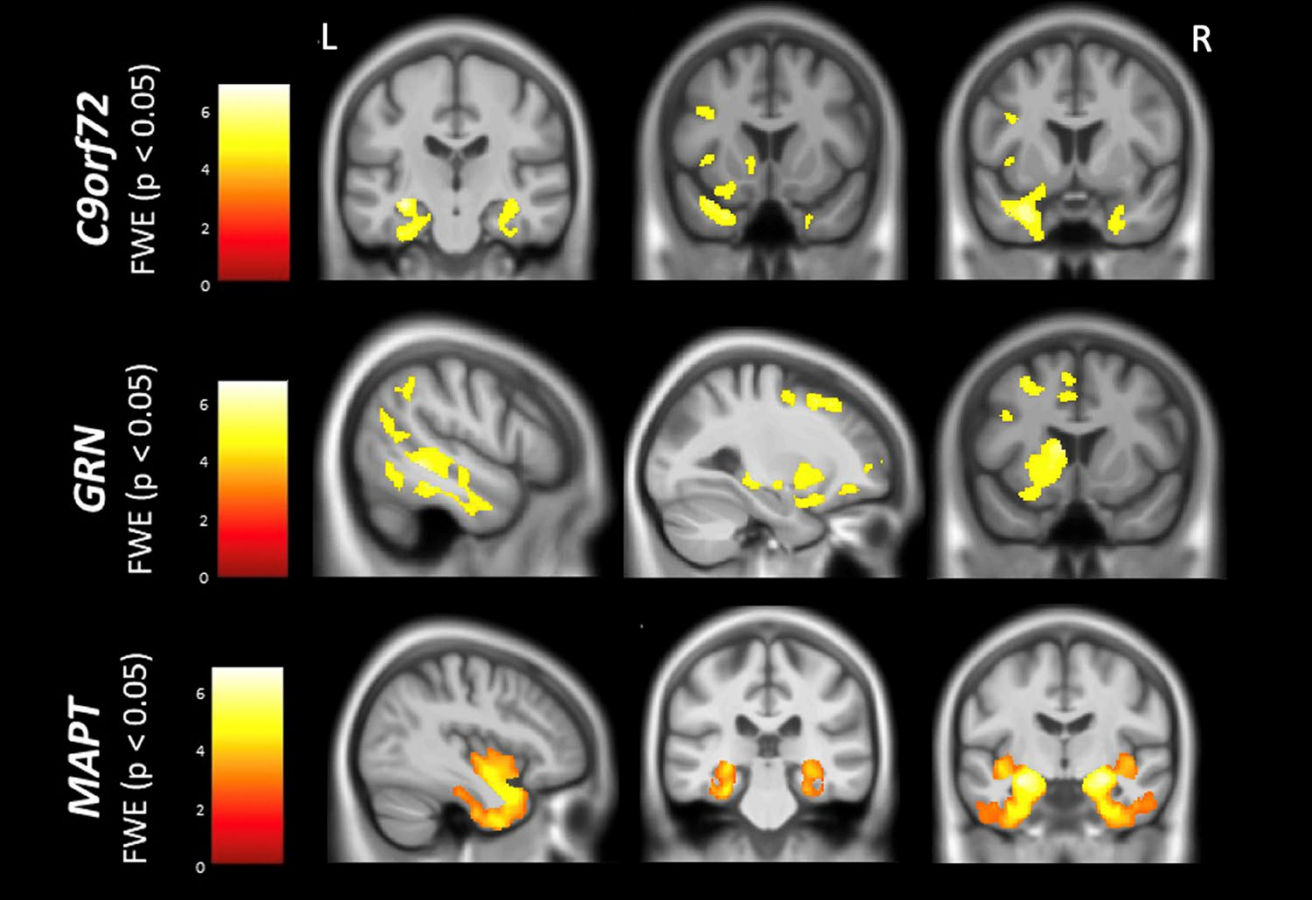
Neural correlates of naming in C9orf72, MAPT and GRN mutation carriers. Results are shown on a study-specific T1-weighted MRI template in MNI space and at *p* < 0.05 for Family-Wise error. Colour bars represent T-values

**Fig. 3:**
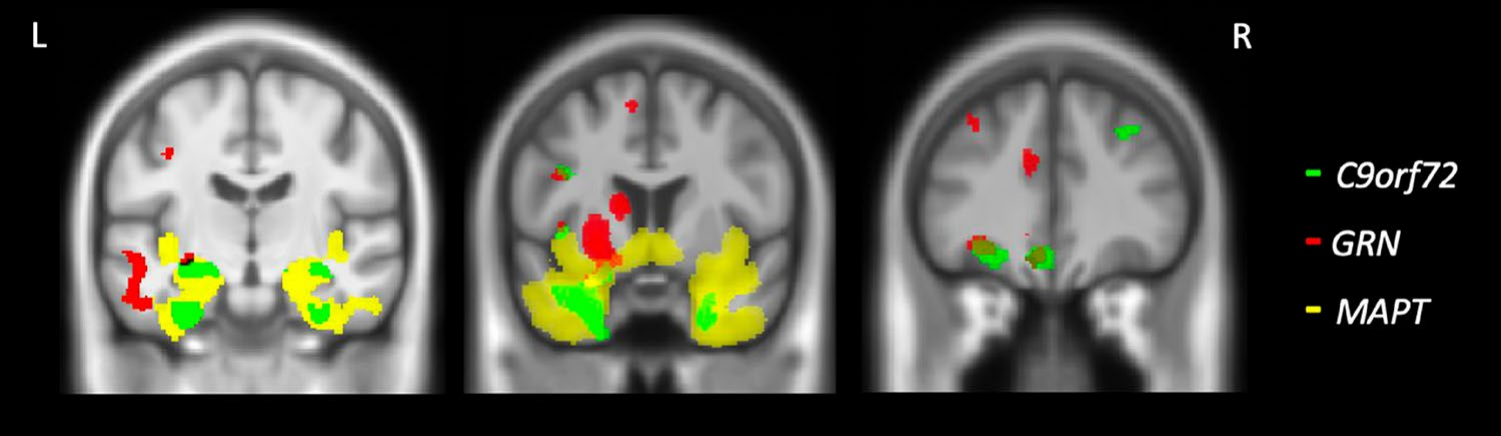
Overlapping neural correlates of naming across the three genetic groups. Comparative results are shown on a study-specific T1-weighted MRI template in MNI space and at *p* < 0.05 for Family-Wise error. The C9orf72 group are shown in green, the GRN group are shown in red and the MAPT group are shown in yellow

The initial VBM analysis model revealed partially overlapping neural correlates of naming in the three genetic groups (Figs. 2, 3, Supplementary Table S4). In *MAPT* mutation carriers, the anterior and medial temporal regions were implicated bilaterally as were the bilateral anterior insular cortices. In *C9orf72* mutation carriers, the anterior temporal structures were also bilaterally involved. However, more widespread correlates of naming were seen in this group, particularly affecting the left hemisphere, in frontal (inferior, middle and superior) and insular cortices as well as the caudate. In *GRN* mutation carriers, correlates were only found within the left hemisphere, but were more distributed than the other two groups, affecting frontal (including premotor and supplementary motor cortices), anterior and lateral temporal, anterior parietal and striatal regions.

Adjusting for disease severity found very similar results in the additional VBM analysis model, although at an uncorrected *p* < 0.001 threshold, with no results found when correcting for multiple comparisons (Supplementary Fig. S1 and Supplementary Table S5): similar neural correlates were seen in each group although with more focal left anterior temporal lobe involvement for the *C9orf72* mutation carriers in this analysis.

## Discussion

In this study, we found that all genetic groups performed significantly worse on the BNT than controls when people were fully symptomatic, but only in the *MAPT* mutation group was naming ability impaired pre-symptomatically, being abnormal in both prodromal and asymptomatic mutation carriers. This highlights that naming performance is significantly impaired in people with genetic FTD, particularly in those with *MAPT* mutations, consistent with the previous literature [3–5, 11]. However, here we demonstrate very early naming change in the *MAPT* genetic group, and with overlapping but distinct neural correlates across the genetic groups: bilateral anterior temporal and anterior insula regions in *MAPT* mutation carriers, with similar temporal lobe involvement as well as more widespread left hemisphere atrophy in *C9orf72* mutation carriers, and only distributed left hemisphere correlates in *GRN* mutation carriers.

The results in *MAPT* mutation carriers are consistent with previous work, where more severe deficits are seen on naming tasks cross-sectionally and the most decline over time is seen compared with both *C9orf72* and *GRN* mutation carriers [4, 19]. We also found that both *MAPT* asymptomatic and prodromal groups performed significantly worse than controls. This finding has not been reported in the literature but is in keeping with previous work showing that *MAPT* mutation carriers have naming deficits before a formal diagnosis of FTD [4, 20]. Our study provides further evidence for subtle cognitive changes at a pre-symptomatic stage. Clinical trials for *MAPT* mutation carriers should consider using naming tasks such as the BNT as a marker for patient selection and outcome measure.

In *MAPT* mutation carriers, focal atrophy within the bilateral anterior and medial temporal lobes was associated with BNT score. The anterior temporal lobe has often been associated with semantic memory, particularly in studies which show that this region is specifically atrophied and hypometabolic in people with the semantic variant of PPA compared with those with Alzheimer’s disease [21]. Symptomatic and late pre-symptomatic *MAPT* mutation carriers are significantly impaired compared to controls on semantic memory tasks, with performance correlating strongly with bilateral temporal lobe volume [22]. Moreover, semantic deficits are suggested to occur with greater frequency in *MAPT* mutation carriers than in *GRN* or *C9orf72* mutation carriers [3–5]. Thus, a core semantic deficit has been put forward as the defective mechanism underlying *MAPT* mutation carriers’ anomia, and our imaging results appear in line with such claims. Moreover, in view of the extremely symmetrical neuroanatomical correlates with the BNT, it appears that both verbal and visual semantics are equally likely to be related to *MAPT* mutation carriers’ poor BNT score.

In *C9orf72* and *GRN* mutation carriers, reduced grey matter volume in the anterior temporal structures was also related to BNT performance. In the *C9orf72* group, these extended to include bilateral hippocampi, whilst in the *GRN* group, these were left hemisphere only. In a recent study of a large cohort of patients, semantic deficits were also found in both *C9orf72* and *GRN* mutation carriers [22]. Thus, semantic memory deficits are likely to underlie at least part of BNT performance. However, our results show that in both *C9orf72* and *GRN* mutation carriers, neuroanatomical correlates of BNT score were more widespread throughout the left hemisphere. Indeed, in *C9orf72* mutation carriers, left-predominant frontal regions and the left caudate were implicated, whilst in *GRN* mutation carriers, left frontal and striatal areas as well as the lateral temporal and parietal cortices were also involved. These findings are consistent with previous studies which have identified different regions to be related to anomia according to the likely linguistic subdomain affecting naming ability. Whilst anterior temporal regions have been found to correlate with naming deficits when semantic impairment is present, such as in the semantic variant of PPA [9, 10, 23], frontal lobe regions may be involved when there is impairment of word generation and motor aspects of speech and language, such as in the non-fluent variant of PPA. These include the inferior frontal lobe, opercular and anterior insula [24–26], as was seen here in both *GRN* and *C9orf72* mutation carriers. In *GRN* mutation carriers alone, more lateral temporal and anterior parietal regions were involved. In the lateral temporal cortex, the superior temporal sulcus was particularly implicated, an area shown to enable audiovisual integration, leading to its implication in semantic processing [27], whilst in the anterior parietal region, classically affected in the logopenic variant of PPA, the angular gyrus was mainly involved, an area usually thought to be associated with semantic processing for both auditory and visual stimuli as well as being involved in concept retrieval and conceptual integration [28]. Finally, the *C9orf72* genetic group showed bilateral frontal involvement, albeit left-lateralised. Previous studies show that executive processes can also be involved in naming, as can be seen in people with bvFTD [3, 9], and it may be that this is playing a role here.

A limitation of the present study is that the nature of incorrect answers on the BNT were not recorded. Error analysis could reveal the contributing processes, for example according to whether the participant gives the superordinate name, a wrong name or no name [29, 30]. Distinct error patterns can be seen as a function of left *versus* right and anterior *versus* posterior temporal lobe atrophy [9]. Our genetic groups showed left/right as well as anterior/posterior differences which could therefore lead to contrasting error patterns. Thus, future work could examine the nature of naming errors to explore whether such patterns differ across genetic groups and correspond to the different anatomical correlates identified. An alternative way to distinguish between the underlying cognitive processes could be to examine the inter-relationship with other linguistic measures. However, specific language tasks were limited in the GENFI neuropsychological battery.

The strength of this study’s results comes from the use of a large cohort of people with genetic FTD, which enabled gene-specific analyses, compared to control group of mutation-negative family members. We were therefore able to find pre-symptomatic naming deficits in *MAPT* mutation carriers and reveal different levels of performance in naming, between the three genetic groups. Different processes underlying naming in each genetic group are suggested by the diverse brain regions which appeared related to naming performance.

## Conclusion

Overall, our findings are consistent with the hypothesis that large‐scale neural network degeneration underlies the impairment of naming ability in genetic FTD, but with different contributory regions in each genetic form. This study highlights the potential use of a simple naming task as an outcome measure for international clinical trials in pre-symptomatic *MAPT* mutation carriers, and in helping differential diagnosis and severity staging by understanding the sources of naming difficulty.

## Supplementary Information

Below is the link to the electronic supplementary material.

Supplementary file1 (DOCX 3195 KB)

## Data Availability

Some GENFI data are available on reasonable request through application to the GENFI Data Access Committee.
