# Supplementary material for: Anomia is present pre-symptomatically in frontotemporal dementia due to MAPT mutations
Source: J Neurol. 2022 Mar 29;269(8):4322–32. doi: 10.1007/s00415-022-11068-0 (PMC9294015; doi:10.1007/s00415-022-11068-0)
Supplement: Supplementary file 1 — Supplementary file1 (DOCX 3195 KB) [file 415_2022_11068_MOESM1_ESM.docx]

**Supplementary Data**

**Bouzigues et al. Anomia is present presymptomatically in frontotemporal dementia due to MAPT mutations**

***Fig. S1.* Neural correlates of naming in *C9orf72, MAPT* and *GRN* mutation carriers with an additional adjustment for disease severity. Results are shown on a study-specific T1-weighted MRI template in MNI space, uncorrected *p*<0.001. Colour bars represent T-values.**

*
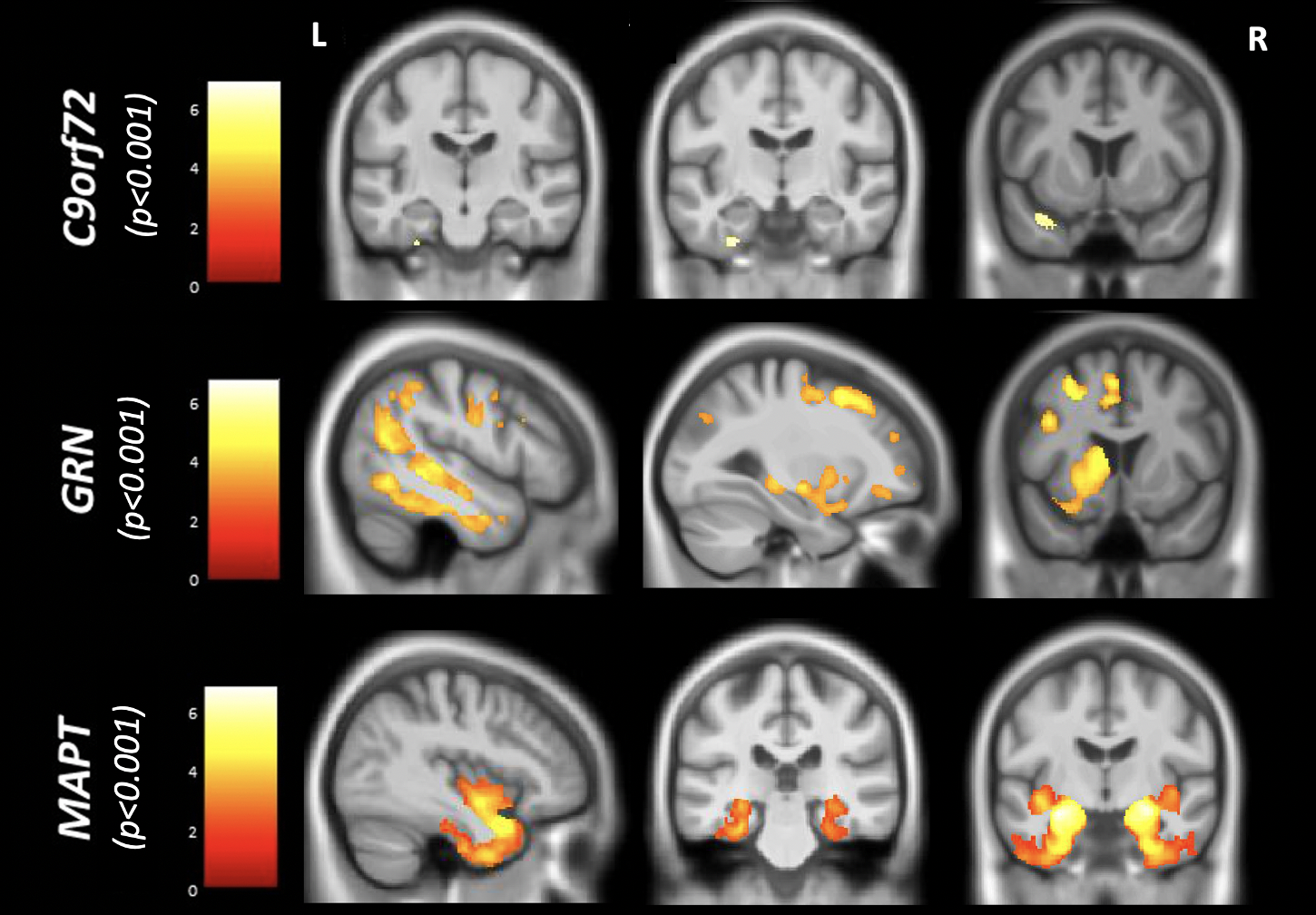
*

***Table S1.* Cumulative frequencies of BNT scores in the control group.**

| **BNT Score** | **N** | **Cumulative Freq** |
| --- | --- | --- |
| 21 | 3 | 1.2 |
| 22 | 2 | 2.0 |
| 23 | 6 | 4.4 |
| 24 | 3 | 5.6 |
| 25 | 14 | 11.3 |
| 26 | 21 | 19.8 |
| 27 | 44 | 37.5 |
| 28 | 54 | 59.3 |
| 29 | 54 | 81.0 |
| 30 | 47 | 100.0 |

***Table S2.* Mean control BNT performance and standard deviations according to age group and sex.**

| **Age group** | **All** | | **Females** | | **Males** | |
| --- | --- | --- | --- | --- | --- | --- |
|  | **N** | **BNT Mean (SD)** | **N** | **BNT Mean (SD)** | **N** | **BNT Mean (SD)** |
| **Overall** | 248 | 27.8 (1.9) | 141 | 27.8 (1.9) | 107 | 27.8 (1.9) |
| 18.1-29.9 | 32 | 27.6 (1.9) | 17 | 27.9 (1.6) | 15 | 27.3 (2.2) |
| 30.0-39.9 | 66 | 27.9 (2.0) | 40 | 27.8 (2.0) | 26 | 28.2 (2.0) |
| 40.0-49.9 | 67 | 28.0 (1.6) | 32 | 27.7 (1.8) | 35 | 28.3 (1.4) |
| 50.0-59.9 | 45 | 27.7 (2.0) | 29 | 27.6 (2.6) | 16 | 27.8 (1.5) |
| 60.0-69.9 | 32 | 27.5 (2.2) | 21 | 28.1 (2.0) | 11 | 26.3 (2.1) |
| 70.0-85.0 | 6 | 26.7 (1.9) | 2 | 27.0 (0.0) | 4 | 26.5 (2.4) |

***Table S3*. Linear mixed model analysis comparing disease groups and controls. For each pairwise comparison, observed contrast, standard error, p values and unadjusted 95% confidence intervals are shown. Pairwise comparisons for which the difference was significant (p<0.05) are shown in bold.**

***
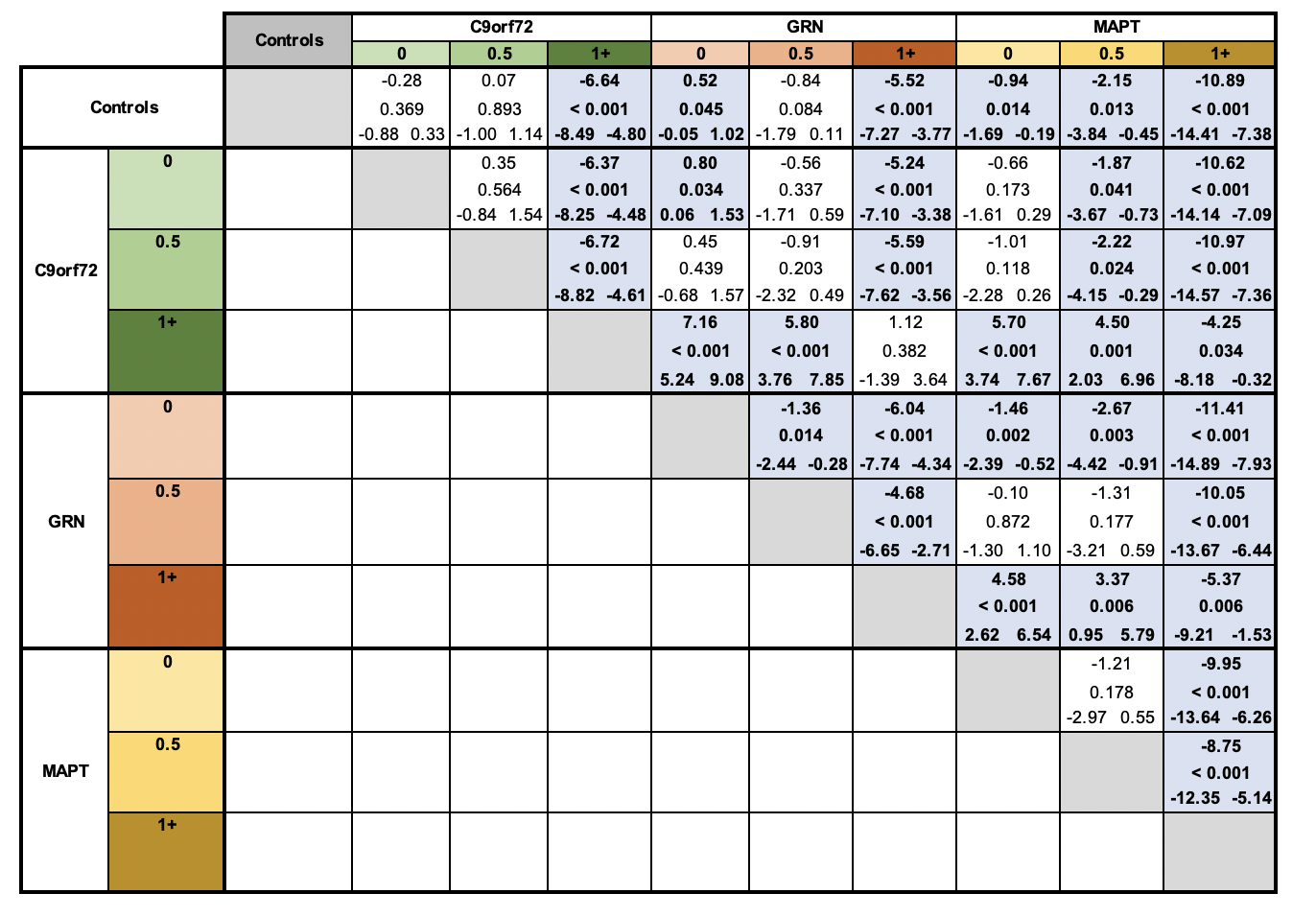
***

***Table S4*. Voxel-based morphometry analysis: grey matter regions correlating significantly with Boston Naming Test score in each genetic group.**

|  |  | | | | | | | | | |
| --- | --- | --- | --- | --- | --- | --- | --- | --- | --- | --- |
|  | **Regions** | **cluster** | **peak** | | | | **coordinates (mm)** | | | |
|  |  | **equivk** | **p(FWE-corr)** | **T** | **p(unc)** | **x** | | **y** | **z** |  |
| ***C9orf72*** | Left hippocampus, fusiform gyrus, amygdala, entorhinal cortex | 6046 | <0.001 | 7.04 | <0.001 | -32 | | -24 | -8 |  |
|  | Left inferior frontal gyrus | 887 | <0.001 | 6.26 | <0.001 | -4 | | 45 | -12 |  |
|  | Right frontal pole and superior frontal gyrus | 242 | <0.001 | 6.14 | <0.001 | 15 | | 64 | 16 |  |
|  | Left middle and superior frontal gyrus | 648 | <0.001 | 5.99 | <0.001 | -24 | | 56 | 6 |  |
|  | Right hippocampus, fusiform gyrus, amygdala, entorhinal cortex | 2437 | <0.001 | 5.87 | <0.001 | 27 | | -8 | -15 |  |
|  | Left inferior temporal gyrus | 572 | <0.001 | 5.79 | <0.001 | -45 | | -44 | -21 |  |
|  | Left middle frontal gyrus | 312 | 0.002 | 5.42 | <0.001 | -40 | | 15 | 30 |  |
|  | Left anterior insula | 122 | 0.002 | 5.41 | <0.001 | -39 | | 10 | 0 |  |
|  | Left fusiform gyrus | 60 | 0.002 | 5.34 | <0.001 | -24 | | -54 | -10 |  |
|  | Right middle frontal gyrus | 148 | 0.003 | 5.33 | <0.001 | 27 | | 62 | 2 |  |
|  | Right superior frontal gyrus | 55 | 0.005 | 5.18 | <0.001 | 26 | | 58 | 12 |  |
|  | Right middle frontal gyrus | 134 | 0.006 | 5.14 | <0.001 | 28 | | 38 | 36 |  |
|  | Left central operculum | 39 | 0.009 | 5.06 | <0.001 | -40 | | -8 | 9 |  |
|  | Right middle frontal gyrus | 48 | 0.009 | 5.04 | <0.001 | 28 | | 18 | 50 |  |
|  | Right temporal pole | 26 | 0.011 | 4.99 | <0.001 | 38 | | 10 | -36 |  |
|  | Left cerebellum exterior | 39 | 0.012 | 4.99 | <0.001 | -18 | | -74 | -40 |  |
|  | Left superior frontal gyrus | 54 | 0.012 | 4.98 | <0.001 | -12 | | 46 | 8 |  |
|  | Left frontal pole | 48 | 0.015 | 4.93 | <0.001 | -14 | | 62 | -9 |  |
|  | Right inferior frontal cortex | 31 | 0.021 | 4.85 | <0.001 | 9 | | 30 | -18 |  |
|  | Left caudate | 95 | 0.022 | 4.83 | <0.001 | -12 | | 15 | 0 |  |

| ***GRN*** | Left superior temporal sulcus and inferior temporal gyrus | 3425 | <0.001 | 7.34 | <0.001 | -58 | -33 | -6 |
| --- | --- | --- | --- | --- | --- | --- | --- | --- |
|  | Left hippocampus | 790 | <0.001 | 6.59 | <0.001 | -21 | -33 | -2 |
|  | Left middle frontal gyrus | 772 | <0.001 | 6.43 | <0.001 | -28 | 15 | 50 |
|  | Left caudate, putamen and accumbens | 3173 | <0.001 | 6.40 | <0.001 | -9 | 14 | 9 |
|  | Left angular gyrus | 415 | <0.001 | 5.70 | <0.001 | -50 | -60 | 18 |
|  | Left supplementary motor cortex | 111 | <0.001 | 5.68 | <0.001 | -4 | 10 | 52 |
|  | Left supplementary motor cortex, superior frontal gyrus | 927 | 0.001 | 5.67 | <0.001 | -4 | 16 | 39 |
|  | Left supplementary motor cortex | 114 | 0.001 | 5.56 | <0.001 | -4 | -4 | 48 |
|  | Left orbitofrontal cortex | 323 | 0.001 | 5.56 | <0.001 | -32 | 42 | -12 |
|  | Left frontal pole | 165 | 0.002 | 5.42 | <0.001 | -20 | 60 | -9 |
|  | Left middle frontal gyrus | 168 | 0.003 | 5.33 | <0.001 | -40 | 14 | 30 |
|  | Left supramarginal gyrus | 173 | 0.003 | 5.33 | <0.001 | -54 | -46 | 44 |
|  | Left angular gyrus | 107 | 0.003 | 5.31 | <0.001 | -39 | -56 | 48 |
|  | Left superior frontal gyrus | 225 | 0.003 | 5.31 | <0.001 | -24 | -4 | 54 |
|  | Left angular gyrus | 44 | 0.005 | 5.19 | <0.001 | -44 | -56 | 32 |
|  | Left superior frontal gyrus | 73 | 0.006 | 5.13 | <0.001 | -22 | 58 | 6 |
|  | Left anterior insula | 41 | 0.015 | 4.92 | <0.001 | -38 | 20 | -2 |
|  | Left premotor cortex | 37 | 0.015 | 4.92 | <0.001 | -36 | -14 | 39 |
|  | Left precuneus | 24 | 0.016 | 4.92 | <0.001 | -8 | -51 | 34 |
|  | Left anterior insula | 27 | 0.027 | 4.79 | <0.001 | -42 | 2 | 4 |
|  | Left middle frontal gyrus | 31 | 0.028 | 4.77 | <0.001 | -26 | 50 | 3 |
| ***MAPT*** | Left temporal pole, amygdala, hippocampus, entorhinal cortex, inferior temporal gyrus and insula | 14502 | <0.001 | 11.32 | <0.001 | -28 | -6 | -18 |
|  | Right temporal pole, amygdala, hippocampus, entorhinal cortex, inferior temporal gyrus and insula | 13592 | <0.001 | 10.91 | <0.001 | 26 | -8 | -15 |
|  | Left fusiform gyrus | 73 | 0.010 | 5.03 | <0.001 | -44 | -44 | -16 |

***Table S5*. Voxel-based morphometry analysis: grey matter regions correlating significantly with Boston Naming Test score in each genetic group, with additional adjustment for disease severity.**

|  | **Regions** | **cluster** | **peak** | | | **coordinates (mm)** | | |
| --- | --- | --- | --- | --- | --- | --- | --- | --- |
|  |  | **equivk** | **p(FWE-corr)** | **T** | **p(unc)** | **x** | **y** | **z** |
| ***C9orf72*** | Left inferior temporal gyrus | 83 | 0.636 | 3.86 | <0.001 | -28 | -14 | -39 |
|  | Left temporal pole | 79 | 0.905 | 3.59 | <0.001 | -34 | 10 | -27 |
| ***GRN*** | Left superior temporal sulcus and inferior temporal gyrus | 8409 | <0.001 | 6.65 | <0.001 | -58 | -33 | -6 |
|  | Left middle frontal gyrus | 3782 | 0.002 | 5.47 | <0.001 | -28 | 15 | 50 |
|  | Left thalamus, hippocampus, caudate, putamen | 4569 | 0.007 | 5.15 | <0.001 | -20 | -34 | -3 |
|  | Left supramarginal gyrus | 881 | 0.053 | 4.66 | <0.001 | -57 | -45 | 39 |
|  | Left supplementary motor cortex, middle cingulate gyrus | 296 | 0.066 | 4.61 | <0.001 | -6 | -6 | 48 |
|  | Left middle frontal gyrus, inferior frontal gyrus | 507 | 0.081 | 4.55 | <0.001 | -40 | 14 | 30 |
|  | Left frontal pole, orbitofrontal cortex, superior frontal gyrus | 292 | 0.201 | 4.30 | <0.001 | -20 | 60 | -9 |
|  | Left orbitofrontal cortex | 273 | 0.318 | 4.15 | <0.001 | -32 | 42 | -12 |
|  | Left precentral gyrus, postcentral gyrus | 503 | 0.342 | 4.12 | <0.001 | -36 | -15 | 38 |
|  | Left and right anterior cingulate gyrus | 100 | 0.415 | 4.05 | <0.001 | 0 | 36 | 4 |
|  | Left middle frontal gyrus, superior frontal gyrus | 226 | 0.443 | 4.03 | <0.001 | -32 | 48 | 26 |
|  | Left precuneus, posterior cingulate gyrus | 110 | 0.642 | 3.86 | <0.001 | -6 | -51 | 34 |
|  | Right cerebellum | 147 | 0.666 | 3.84 | <0.001 | 45 | -72 | -50 |
|  | Left middle frontal gyrus, precentral gyrus | 148 | 0.67 | 3.83 | <0.001 | -44 | 6 | 51 |
|  | Left superior frontal gyrus | 192 | 0.737 | 3.78 | <0.001 | -22 | 58 | 6 |
|  | Right occipital pole | 70 | 0.791 | 3.73 | <0.001 | 26 | -98 | 15 |
|  | Left inferior frontal gyrus | 119 | 0.804 | 3.71 | <0.001 | -39 | 42 | 4 |
| ***MAPT*** | Left amygdala, hippocampus, entorhinal cortex, temporal pole, inferior temporal gyrus, insula and fusiform gyrus | 12258 | <0.001 | 9.39 | <0.001 | -30 | -4 | -20 |
|  | Right amygdala, hippocampus, entorhinal cortex, temporal pole, inferior temporal gyrus, insula and fusiform gyrus | 12482 | <0.001 | 8.90 | <0.001 | 26 | -8 | -15 |
|  | Left insula | 1055 | 0.006 | 5.18 | <0.001 | -3 | 6 | -2 |
